# Metastatic renal cell carcinoma presenting as a duodenal mass

**DOI:** 10.1002/ccr3.6036

**Published:** 2022-07-22

**Authors:** Spyridon Vrakas, Epameinondas Skouloudis, Georgios Koutoufaris, Kassiani Manoloudaki, Dimitrios Karapiperis, Vasilis Xourgias

**Affiliations:** ^1^ Department of Gastroenterology Tzaneion General Hospital Piraeus Greece; ^2^ Department of Pathology Tzaneion General Hospital Piraeus Greece; ^3^ Department of Gastroenterology Vrinnevi General Hospital of Norrkoping Norrkoping Sweden

**Keywords:** duodenum, metastasis, renal cell carcinoma

## Abstract

We report a case of renal cell carcinoma metastasis to the duodenum.

## Graphical abstract

Renal cell carcinoma (RCC) is a common cause of cancer‐related mortality worldwide. It rarely affects the gastrointestinal tract and should be considered in the differential diagnosis in patients with iron deficiency anemia, a mass in the GI tract, and a history of RCC.

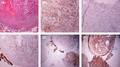

Α 55‐year‐old woman was admitted to our hospital for iron deficiency anemia. From medical history, she underwent right radical nephrectomy due to clear cell carcinoma 8 years ago. The patient underwent colonoscopy without pathological findings. Esophagogastroduodenoscopy revealed a mass in the second part of the duodenum (Figure 1), and biopsies were taken. Histopathology examination showed sheets of clear cells (Figure 2). Immunohistochemistry was positive for CD10, PAX8, EMA, Cam5‐2, and vimentin, while CK7 was negative (Figure 2). Diagnosis of metastatic renal cell carcinoma (RCC) was confirmed. Computer tomography showed metastases to the lungs, liver, brain, and duodenum. The left kidney revealed no abnormalities. Oncology department was consulted, and the patient was planned for immunotherapy.

**FIGURE 1:**
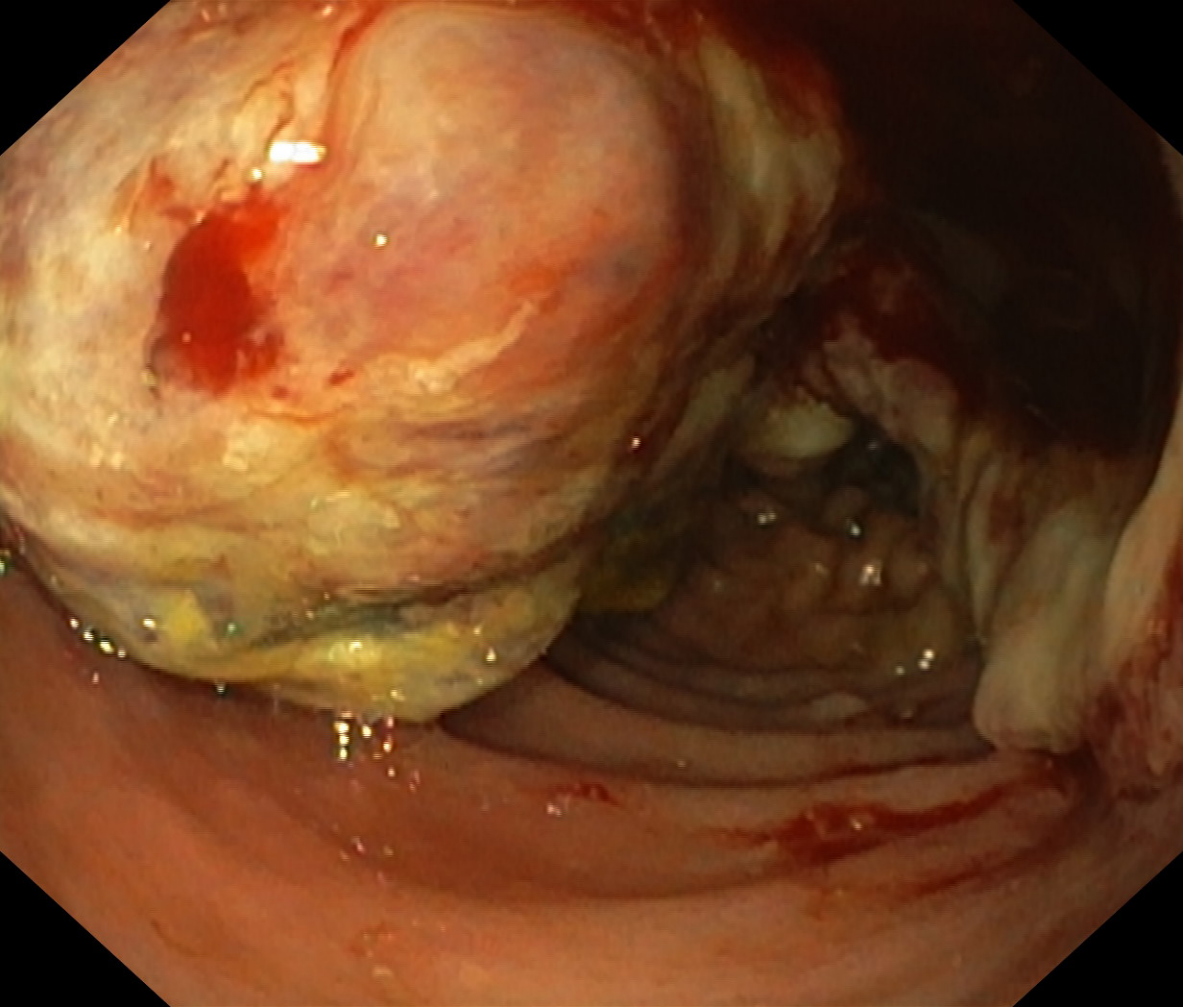
Photograph of a 55‐year‐old woman with a mass in the second part of the duodenum

**FIGURE 2:**
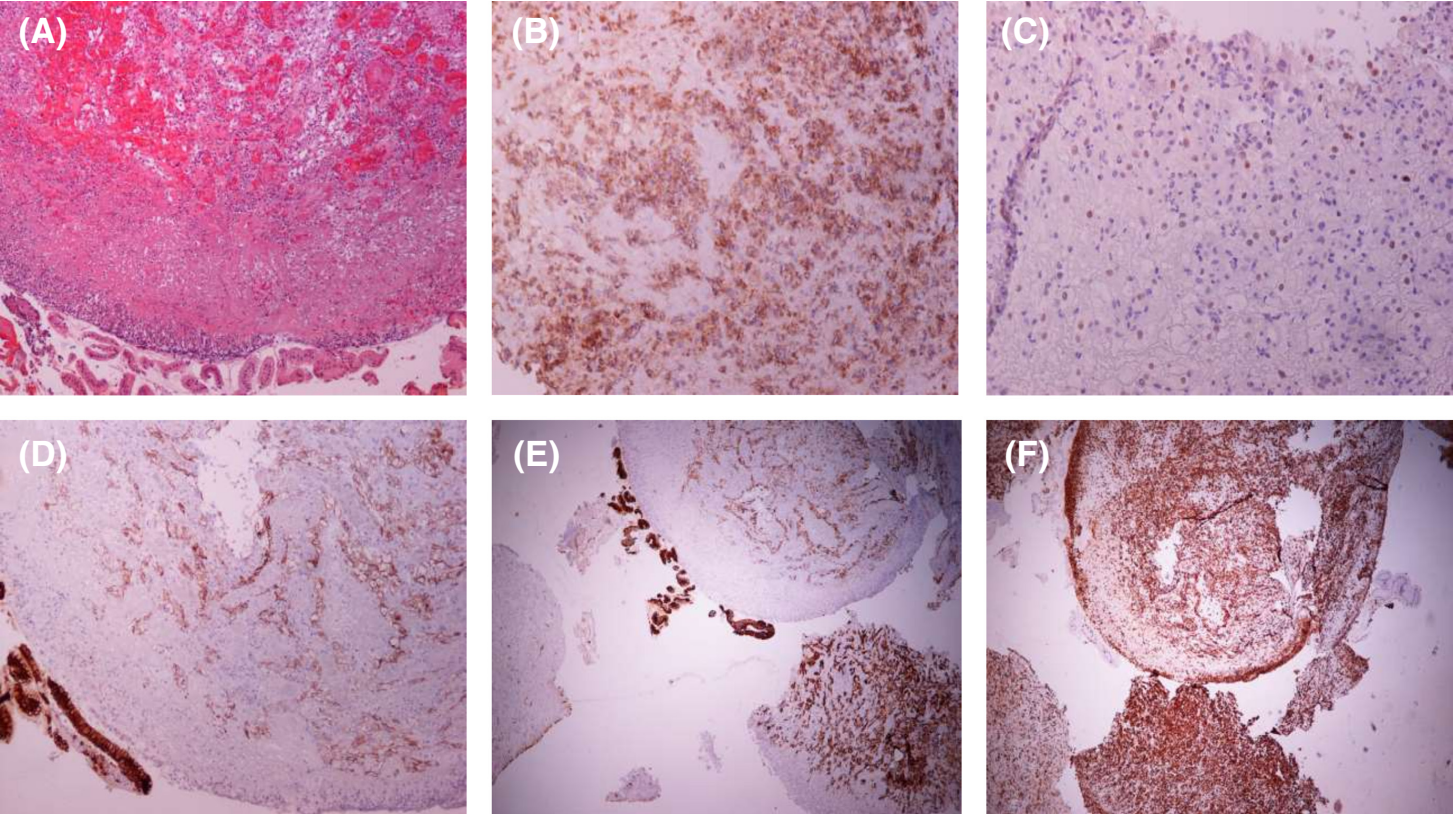
Photographs of (A) histopathology examination showing sheets of clear cells, while immunohistochemistry was positive for (B) CD10 (C) PAX8 (D) EMA (E) Cam5‐2, and (F) vimentin

Renal cell carcinoma is the most common type of renal carcinomas in patients above 50 years.^1^ Recurrence is unpredictable and rarely affects the gastrointestinal tract. Metastasis to GI tract is more common in small intestine and the time from resection of the primary tumor until the diagnosis of metachronous metastasis varies.^2^ Therefore, long‐term follow‐up is needed. Renal cell carcinoma metastasis should be considered in the differential diagnosis in patients with iron deficiency anemia, a mass in the GI tract, and a history of RCC.

## AUTHOR CONTRIBUTIONS

VS, ES, GK, MK, DK, and XV contributed to the writing and approval of the final manuscript.

## CONFLICT OF INTEREST

None declared.

## CONSENT

Written informed consent was obtained from the patient to publish this report in accordance with the journal's patient consent policy.

## Data Availability

Data supporting the findings of this study are available from the corresponding author on request.
